# Should farmers apply fertilizer according to when their daffodils are in flower? Utilizing a “farmer‐science” approach to understanding the impact of soil temperature on spring N fertilizer application in Wales

**DOI:** 10.1111/sum.12503

**Published:** 2019-04-12

**Authors:** Felicity Crotty, Heather McCalman, Huw Powell, Sue Buckingham, Christina Marley

**Affiliations:** ^1^ Institute of Biological, Environmental and Rural Sciences Aberystwyth University Aberystwyth UK; ^2^ Game & Wildlife Conservation Trust Allerton Project Loddington UK; ^3^ Felicity Crotty Royal Agricultural University Cirencester UK; ^4^ Quality Meat Scotland The Rural Centre Newbridge UK; ^5^ Natural England West Midlands Team Worcester UK

**Keywords:** citizen science, daffodils, nitrogen fertilizers, seasonality, soil temperature, The PROSOIL project

## Abstract

Perennial ryegrass starts growing when soil temperatures reach 5.5°C for five consecutive days; applying N fertilizer before this risks environmental losses. To test whether daffodil flowering signified when to apply N fertilizer, farmers volunteered to take part in a citizen science study. The PROSOIL project used a “citizen science”, participatory approach to create farmer‐informed science, aiming to increase awareness of the importance of soil health. In 2014, over 300 farmers completed a “*How do you manage your soil*” survey. The survey included a question on the use of daffodils (*Narcissus* spp.) to indicate the best time to apply the first nitrogen fertilizer of the season, based on anecdotal feedback from farmers involved in the PROSOIL project. The survey recorded 7% of farmers based their first fertilizer application on when daffodils flowered. To increase farmer awareness of soil temperatures, we provided them with soil thermometers, held workshops and hosted interactive stands at agricultural events in 2014. In autumn 2014, farmers planted daffodil bulbs of the same variety, across Wales, and monitored soil temperatures. Farmers returned postcards once their daffodils were in flower, noting the soil temperature. An assessment of whether daffodil flowering date could indicate when to apply N fertilizer was made. Overall, in spring 2015, daffodils flowered when soil temperature was 6.4(±0.35)°C, suggesting daffodil flowering date is a more reliable indicator for fertilizer application, than first hypothesized. Findings show a scientific validation of local knowledge, regarding the use of daffodils to indicate the “not‐before” date for the first N fertilizer application.

## 1 INTRODUCTION

Over the past few decades, food production (livestock and arable) has started to plateau, whilst world population continues to rise (Conway & Toenniessen, 1999). Consequently, feeding the world will become a growing problem that needs to be solved sustainably (e.g. sustainable intensification Garnett et al., 2013). Grassland soils provide a number of key ecosystem services – including water and nutrient cycling, climate regulation, a habitat sink for predatory invertebrates that assist in agricultural pest control, as well as for food production (Murray, Crotty, & Van Eekeren, 2012). Within the European Union (EU), around 30% of food is obtained from animal products (Borlaug, 2002). Globally, increased meat consumption per capita and population growth have resulted in an overall increase in meat production and consumption (Vranken, Avermaete, Petalios, & Mathijs, 2014). In the United Kingdom (UK), agricultural land area covers around 17 million hectares (70% of the land area). In Wales, in 2015, agricultural land area covered around 88% of the country (1.75 million hectares), of which 75% was permanent pasture grassland (Armstrong, 2016). It is vital for sustainable production that the quality and output of these permanent pasture grasslands is maintained.

Welsh farming is heavily focused on livestock (51% of farms) and livestock products (35%), with 40% of the UKs sheep and cattle being farmed in Wales across only 10% of the agricultural land area for the United Kingdom (Armstrong, 2016). Livestock need a consistent supply of high‐quality forage (or silage) to maintain economically viable production and mineral N fertilizer has traditionally been used to achieve this (Butler, Biermacher, Kering, & Interrante, 2012). Grass productivity is highly dependent on nitrogen (N) inputs, and there is concern that management to maximize food production could cause a decline in the overall habitat quality, including an increase in greenhouse gas (GHG) emissions (Horrocks, Dungait, Heal, & Cardenas, 2014). Applications of N fertilizer to grassland have shown an overall decline between 2000 and 2015, to an average of 56 kg ha^−1^ (DEFRA, 2016). This decline is largely due to a greater environmental awareness and pressure to reduce soil nutrient balances; as well as cost savings (with prices for ammonium nitrate (UK) increasing from around £100 per tonne 2001/2 to over £300 per tonne 2011/12 (AHDB Dairy, 2017)). However, the Department for Environment, Food and Rural Affairs Nutrient Management Guide (DEFRA RB209, 2010) recommends between 70 – 310 kg N ha^−1^ year^−1^ for grass silage in the United Kingdom; for grazing, it recommends between 30 – 270 kg N ha^−1^ year^−1^. Timing of fertilizer application in the spring is crucial, as ryegrass does not typically begin to grow until soil temperatures reach 5.5°C for five consecutive days (Frame, 1992). The addition of mineral fertilizer has been shown to stimulate denitrification and greenhouse gas N_2_O losses where soil temperatures were cooler than 6°C (Ellis, Yamulki, Dixon, Harrison, & Jarvis, 1998). Therefore, applying nitrogen too early, prior to grass growth, could lead to losses through leaching, run‐off, denitrification and ammonia volatilization (DEFRA, 2010).

Farmers have a wealth of local knowledge gained from their experience of living and working the land often, but not always, within the same rural community over many generations. This local knowledge (gained through routine, site‐specific practices, produced in association with the wider objectives – farming – or identity of that particular social group – livestock farmers – (Morris, 2016)) shows an understanding of the local environment, and this knowledge can influence scientific research design and questions. This builds on a concept that for research to be more equitable, it needs to include academic and relevant non‐academic forms of knowledge, a concept initially proposed by Kloppenburg (1991). Citizen science, defined as “public participation in organized research efforts” (Dickinson & Bonney, 2012), is often used to gather large quantities of data across large geographic scales and hard‐to‐access places. Citizen science methodologies vary from counting abundances, mapping resources, providing real‐time monitoring or pollution detection. All of these activities promote public input and engagement building on scientific knowledge and encouraging public action (McKinley et al., 2017). The success and sustainability of agricultural citizen science projects depends on volunteer farmers who are motivated to contribute their time, energy and skills (Beza et al., 2017). Engaging “citizen scientists” in data collection within their own community provides a link between academics and the community that they are trying to engage. Farmers value knowledge delivered by peers or in discussion rather than prescriptive farmer–scientist knowledge exchange (Fazey et al., 2013; Ingram, 2013; see Fry and Thieme; Wick et al., this issue). A fundamental component of citizen science is the reciprocal relationship between the citizens contributing to the project and the academics – without the citizens contribution a participatory research project cannot be performed to the extent that it is (Pollard, Roetman, & Ward, 2017).

The PROSOIL project was set up to promote maintaining healthy soils on livestock farms in Wales and created a network of farmer groups across Wales to facilitate peer‐to‐peer learning, with knowledge exchange as a key deliverable to act as a conduit for citizen science activities. Engaging farmers through relevant farmer identified topics can lead to an increase in understanding of the conflicting issues (e.g. shorter‐term productivity gains versus longer‐term soil health improvement) and greater dissemination across the community. Through active collaborative farmer engagement, the PROSOIL project connected with over 900 people within the farming community. Over the timescale of the PROSOIL project, farmers provided the PROSOIL project team with questions that they wanted further information on; this led to the development of citizen science (farmer‐science) projects.

One aspect of farmer participation within the PROSOIL project was the completion of a farmer survey, which assessed how individual farmers across Wales monitored the health of their soil and considered the effect agricultural management had on their soils (spring to summer 2014). The survey included a question on the use of daffodils (*Narcissus* spp) to indicate the best time to apply the first N fertilizer application of the season; this was included because farmers involved in the PROSOIL project had suggested daffodil flowering was an approach they used. The daffodil study reported here has two goals: (a) to gather scientific data, alongside citizen science data to compare daffodils efficacy in determining the best time to apply N fertilizer at the beginning of the season; and (b) farmer education (similar to the study described by Polidoro & Clement, 2018, and in agreement with Turrini, Dörler, Richter, Heigl, & Bonn, 2018). The lack of scientific evidence for daffodil flowering date to be a viable indicator for fertilizer application, and the importance of timing of N fertilizer on utilization and losses to the environment highlighted a need for further studies to investigate the linkage between daffodil flowering dates and the correct time to apply N fertilizer. Therefore, the aim of this daffodil study was to engage with farmers and to increase awareness of the relationship between soil temperatures and timings of fertilizer application. Owing to the cost of fertilizer and potential for environmental damage if N fertilizer is applied too early, as well as the reduced growth of ryegrass if N fertilizer is applied too late, understanding when to apply fertilizer can significantly impact on the sustainability of farm businesses.

Daffodils are considered to herald the arrival of spring, reappearing every year after lying dormant over the winter (Kingsbury, 2013). Daffodils require a cold period prior to normal growth resuming in the spring; the flowering date is dependent on spring soil temperatures being sufficiently high (Hanks, 2003). Wales has a strong cultural association with the daffodil; the flower is one of its national symbols, often worn on the nation's saint day (St David, 1 March) which coincides with the first day of spring, according to the meteorological calendar (Kingsbury, 2013). These strong cultural beliefs may have increased the symbolism and influence of the flowering of the daffodil, leading to Welsh farmers being more aware of flowering date. The pattern of flowering is similar to that of other plants in spring, with the increasing soil temperatures causing daffodils to produce flowers, usually occurring before air temperature reaches 15°C (Hanks, 2003). Therefore, daffodil flowering does have the potential to be used as a proxy measure for when grass pasture begins to actively grow, although this relationship had yet to be investigated. The findings presented here tested the scientific null hypothesis that there was no relationship between daffodil flowering date and the correct time to apply the first N fertilizer to grassland in that season.

## 2 METHODS

### 2.1 The PROSOIL project farmer survey 2014

The PROSOIL project *“How do you manage your soil”* farmer survey (2014) was set up to understand how farmers across Wales managed their soils. General questions regarding topography, farm type and soil type were asked along with what soil tests were performed on farm, the timing of fertilizer application and what the farmer considered to be the most important points or problems related to maintaining a healthy soil. The survey was questionnaire‐based, written to meet the requirements of Aberystwyth University (AU) guidelines for research involving human participants. The surveys were as follows: (a) sent out to farmers who had volunteered to join the PROSOIL project mailing list; (b) made available to farmers visiting the PROSOIL stand at the Royal Welsh Agricultural Show in 2014; and (c) online survey; reaching up to 900 farmers across the country, with 304 respondents.

### 2.2 On‐farm daffodil study

In spring and summer 2014, five PROSOIL project “regional development groups” were set up across Wales (located in the southwest, southeast, mid, northwest and northeast Wales). Each group had between 8 and 12 core farmer members who had indicated their interest to form one of these farmer groups so that they could actively participate in peer‐to‐peer learning related to healthy soil management. As part of their group activities, workshops were held in relation to the importance of soil temperature and the timings of fertilizer application. Each group member (67 farmers) was given a bag of daffodil bulbs (containing two to five bulbs of the early‐flowering variety “Tamara”), instructions for correct planting procedures, a soil thermometer and prepaid postcards to fill in and send back to the PROSOIL project team when the daffodils were in flower. It was important that all farmers involved in the study were given the same variety of daffodil bulbs so that results could be compared across Wales. Farmers taking part in this study were given instructions to plant bulbs before November, in a 10‐cm deep hole, with the shoot facing upwards and in area that was not going to be disturbed over the winter but that would be seen or visited regularly. When planting two or more bulbs, farmers needed to allow a space twice the width of a bulb between each. Bulbs needed to be covered with soil and allowed to grow undisturbed for around 3 months. In the “spring”, soil temperature measurements were taken at a depth of 10 cm using a thermometer, when the daffodil plant was in flower. The farmers were asked to return a prepaid postcard (or e‐card reminder) to the PROSOIL project with a record of the soil temperature when the first daffodil had flowered and the date.

### 2.3 Soil temperature monitoring

A weather monitoring station is located at the Gogerddan campus of the Institute of Biological, Environmental and Rural Sciences (IBERS), Aberystwyth University, Wales (52°25′59″N, 4°1′26″W) for the last 50 years. Soil temperatures are monitored at 10‐cm soil depth and recorded hourly (IBERS site). The weather data were supplied by The Meteorological Office, UK. As described in Crotty, Fychan, Scullion, Sanderson, and Marley (2015) soil temperatures were typical of a temperate European climate, with the 50‐year average of 3.8°C in winter and 16.8°C in summer. Daily soil temperatures from January 2015 to April 2015 were downloaded to assess the timing of fertilizer application for the mid‐Wales area. To provide an understanding of temperatures across Wales, data were also downloaded from COSMOS UK (2017), to include two other sites of weather data, one at Bickley Hall (BH), northeast Wales and one near South Wales (Rothamsted North Wyke (RN) – agricultural grassland research site). These two sites were chosen due to their location, habitat and availability of data over the time period. Soil temperature is measured using a Hukseflux STP01 soil temperature profile at 2, 5, 10, 20 and 50 cm intervals; although only 10 cm temperature data are used within this work.

## 3 RESULTS

### 3.1 The PROSOIL project farmer survey 2014

A total of 299 farmers responded to the PROSOIL project 2014 survey (304 responses received over all; representing approximately 1% of Welsh farmers) with similar numbers from each of the regional development groups across Wales. These farmers were representative of lowland (36%) and upland (43%) farms, with 16% identifying as both. The majority surveyed were conventional livestock farmers (76%), whilst 16% were mixed farmers (livestock and arable crops) with <1% farming arable crops only; 8% of those responding were organic livestock farmers. Farming on soil that they classified across all major texture classes (heavy (clay) (33%), medium (loamy) (49%), light (sandy) (13%) and peaty (5%)). Farmers responded from across Wales, covering the major soil types – brown podzolic, ferric stagnopodzols, brown earths and cambic stagnogley/stagnohumic gley soils (Soil Survey of England and Wales, 1983). Sheep were the most common livestock farmed – 47% were combined sheep and beef farmers; 13% sheep only farmers; 4% dairy and sheep enterprises; whilst 5% identified as sheep, beef and dairy. Dairy enterprises were the next most common responders with 13% dairy only and 5% dairy and beef enterprises.

When the respondents were asked when they decided to apply the first N fertilizer of the season, 4% stated they followed the advice of a consultant; the majority stated they decided on applications based on the weather forecast (19%), soil conditions (17%) or a combination of these two factors (31%), as well as time available (12%). Almost 16% of respondents stated they applied fertilizer in relation to soil temperature; however less than half of these respondents (46%) stated in an earlier question that they had monitored soil temperature in the last three years (therefore over half were referring to a perceived warming of the soil). When asked how they decided how much fertilizer to apply, most farmers stated they applied the same every year (29%) or used DEFRA RB209 guidelines (19%); 16% used results from soil testing as a guide for fertilizer application. When asked specifically about daffodil flowering times, 7% of respondents stated that they related their N fertilizer application to daffodil flowering.

### 3.2 On‐farm daffodil study

**Figure 1:**
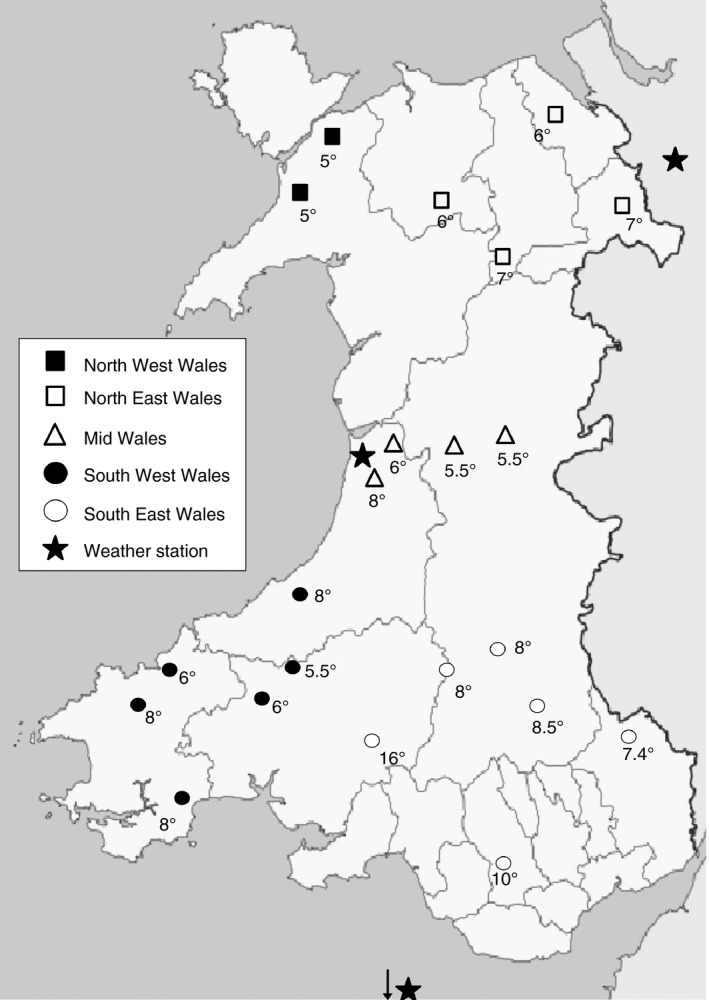
Soil temperatures recorded by farmers across Wales when the daffodils provided by PROSOIL were flowering (°C)

Daffodils were given to 67 farmers from the “regional development groups”, so that there was an even distribution of daffodils planted across Wales. From these, about half (33) of the farmers provided results stating whether the daffodil was in flower and what the soil temperature was at this time. All farmers that were given daffodils were contacted via email during spring to remind them to send in their results. Two thirds of the data provided could be plotted on the map (Figure 1) showing the distribution of daffodil flowering across Wales in spring 2015. A few respondents sent the postcards back early, when the daffodils were only starting to sprout shoots, or did not include a temperature recording on their postcard. Farmer data provided from the “regional development groups”, showed that temperatures are colder in northwest Wales at daffodil flowering (5°C) than in the southeast (9.6°C average); whilst the temperatures across southwest to mid to northeast were intermediate (6.5°C average; Figure 1). Initial interpretation of the data suggests that daffodil flowering date is (for the majority) after soil temperatures reach 5.5°C (the threshold for grass growth). There was an overall average temperature of 6.4°C (± 0.35) across Wales in spring 2015, for when daffodils flowered.

### 3.3 Soil temperature monitoring

**Figure 2:**
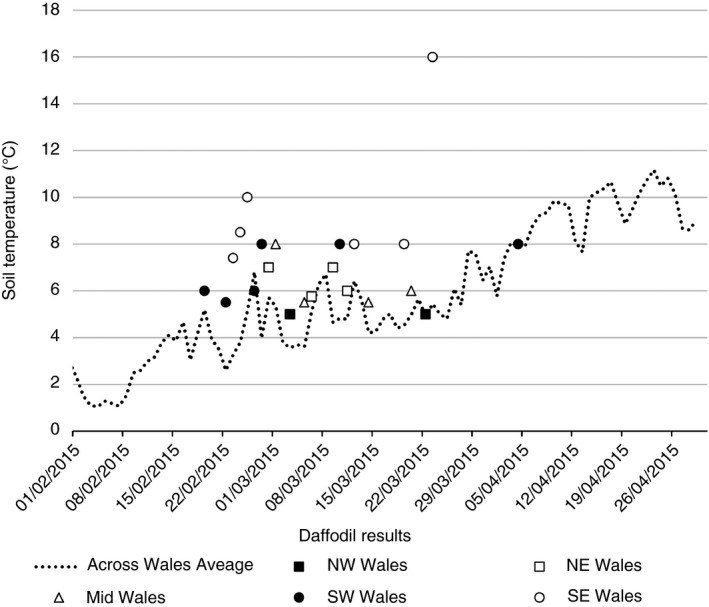
Soil temperature records for when daffodils began to flower in relation to soil temperatures provided by long‐term monitoring stations

In early January, (1–15, 2015) at both IBERS and RN the majority of days had soil temperatures above 5.5°C. However, soil temperature did not consistently exceed 5.5°C until after the 19 February 2015 for these two sites (IBERS and RN), whereas the BH weather station did not report soil temperatures above 5.5°C until the 7 March 2015. Additionally, all three sites did not have five consecutive days with temperatures above 5.5°C until later in March (11 for RN; 30 for BH and 1 April for IBERS). When these temperatures are related to the results provided by farmers, there is some correlation (with the first recorded daffodil flowering being in SW‐Wales on the 19 February (Figure 2). Due to regional variations, the relationships do not correlate fully; however, daffodil flowering did not occur when the soil was below 5°C.

## 4 DISCUSSION

### 4.1 Outcomes of local knowledge and citizen science

The overall findings indicate experiential knowledge acquired through practical experience (Krzywoszynska, 2016) by the farmers involved in both the survey and daffodil study. Results also contradicted the scientific null hypothesis that there was no relationship between daffodil flowering date and the correct time to apply the first N fertilizer to grassland in that season. Farmers rely heavily on informal knowledge, often “learned by doing” rather than from science, and that is often undervalued (Šūmane et al., 2018; see Fry and Thieme, this issue), particularly in an academic context. The results are, to some extent, a scientific validation of farmers’ knowledge, demonstrating that farmers can use daffodil flowering as an indicator to spread fertilizer on pasture in Wales in early spring. Farmers could use daffodil flowering as a “not‐before” date for the first N fertilizer application; however, only monitoring soil temperatures would confirm this in relation to their particular location. It is also worth noting that climate change may alter this assumption if the predicted climate extremes will cause soil temperatures to fluctuate more widely in the future. If farmers waited to apply fertilizer until daffodil flowering, the likelihood is that the fertilizer application would not be lost (through leaching or GHG emissions), whereas if the fertilizer was applied earlier than the daffodil flowering period, it would most likely be lost from the system and not available for plant uptake.

### 4.2 Response rates and data acquired

From the “*How do you manage your soil*” farmer survey, carried out in 2014, we obtained respondents from approximately 1% of Welsh farmers (Armstrong, 2016), therefore providing a representative sample of farming in Wales. The aim of the PROSOIL project was to work with farmers to increase awareness of the importance of soil health and overall, an evaluation of the PROSOIL project by an external evaluator found that farmers involved in the project had increased their knowledge and interest in soil. However, it is worth noting that at the time of the initial survey, most respondents had not previously been involved with the PROSOIL project (80% of respondents) as the survey was mainly completed during the Royal Welsh Agricultural Show 2014. Therefore, the results were not based on a self‐selected group of individuals who had already expressed an interested in soil health, increasing the breadth of scope to capture all farming opinions. At previous workshops during the PROSOIL project, farmers had mentioned that they used daffodil flowering as an indicator for timing of fertilizer application; therefore, this question was included in the 2014 survey. Receiving responses from half the participants of the daffodil study is regarded as a successful response rate for this type of participatory study (Moser & Kalton, 2017), because of the active farmer participation needed, firstly in actively participating in the planting of daffodils in the autumn and then reporting their study data the following spring. Two thirds of these data could be plotted on the map (Figure 1) showing the distribution of daffodil flowering across Wales in spring 2015. The majority of Narcissus species are synanthous (flowering stem and leaves appearing at the same time) (Hanks, 2003). Therefore, all results from farmers were included on the soil temperature map that stated the daffodil was either in flower or bud and had temperature data supplied with it. Although daffodils need to be planted in the autumn and a cold requirement is necessary for bud break and shoot extension, mild winters in the United Kingdom satisfy this, with air temperatures at 9°C being sufficiently cool if occurring for around 120 days (Rees & Hanks, 1996). Overall, in spring 2015, daffodils were reported by farmers as flowering across Wales when the soil temperature was 6.4°C (± 0.35), flowering temperatures ranged from 5 to 16°C and followed the daily monitored soil temperatures from the three different monitoring points (Figure 2).

### 4.3 Implications

Ellis et al. (1998) found gaseous losses of N still occurred following fertilizer or manure addition when soil temperatures were <6°C. Vogeler, Lucci, and Shepherd (2015) found the mistiming of N fertilizer application had a large risk of direct loss through leaching. However, if soil and climatic conditions are ideal, N fertilizer application in the spring reduces the risk of GHG emissions, compared with autumn/winter applications (Bourdin, Sakrabani, Kibblewhite, & Lanigan, 2014). In 2012, GHG emissions from agriculture in Wales was around 13% (6,124 Mt CO_2_ eqivalent similar to transport) of total GHG emissions, whereas agriculture in England produced around 7% of all GHG emissions (Thomas, 2014). Welsh Government recently (2017) completed a consultation considering a review of Nitrate Vulnerable Zones in Wales – areas at risk from agricultural nitrate pollution. The timing of fertilizer application may reduce the risk of pollution on farm from leaching through the soil profile and as surface run‐off to watercourses. Overall, supplying N fertilizer to plants that are actively growing is a key component for effective soil and land management and improved livestock performance and profitability. In the future, further farmer surveys could be used to investigate the use of daffodils as indicators of the best time to apply their first spring fertilizer application, to determine whether, possibly as a result of this participatory action research, a greater proportion of farmers now apply N in relation to daffodil flowering.

## 5 CONCLUSIONS

The PROSOIL project was an example of participatory research that bridges the gap between the scientific and farming community. For this example in Wales, it reached over 900 farmers and increased awareness of the importance of soil health. This daffodil study fostered a relationship between the farming and academic communities, providing scientific validation to local farmer knowledge, demonstrating active knowledge exchange between academics and farmers. This participatory research model could be built on as an example of how to create change through industry‐science collaborations. This study, connecting academia to the farming community, was influenced by farmers from inception and highlights the importance of participatory action initiatives in science (see Stoate et al., this issue). The daffodil planting engaged farmers and increased their interest in soil management, encouraging them to share information, as was evidenced through attendance at soil workshops held throughout spring 2015 (presenting results from the daffodil study as they were being gathered) and improved awareness of the importance of soil temperature in timing of N fertilizer application. The link between daffodil flowering and N fertilizer application date was not considered accurate by the research community, as there were no peer‐reviewed publications indicating this link available. Through the results provided by the farmers involved in this study and the soil temperature monitors, the findings of this study indicate that daffodil flowering could be used as a “not‐before” date for the first N fertilizer application, particularly if used in conjunction with site‐specific soil temperature monitoring. Overall, this study has shown that it is possible to provide scientific validation of local knowledge and increase awareness of the importance of the timing of first N fertilizer applications, through participatory research by the farming and scientific communities.

## References

[sum12503-bib-0001] AHDB Dairy (2017). Fertiliser prices. Retrieved from http://dairy.ahdb.org.uk/resources-library/market-information/farm-expenses/fertiliser-prices/#.W1sqYdJKiUl

[sum12503-bib-0002] Armstrong, E. L. (2016). Research Briefing: The farming sector in Wales. In: *National Assembly for Wales: Research Service* Retrieved from http://www.assembly.wales/research%20documents/16-053-farming-sector-in-wales/16-053-web-english2.pdf Last Accessed 8 March 2019

[sum12503-bib-0003] Beza, E. , Steinke, J. , van Etten, J. , Reidsma, P. , Fadda, C. , Mittra, S. , … Kooistra, L. (2017). What are the prospects for citizen science in agriculture? Evidence from three continents on motivation and mobile telephone use of resource‐poor farmers. PLoS ONE, 12, e0175700. 28472823 10.1371/journal.pone.0175700 PMC5418078

[sum12503-bib-0004] Borlaug, N. E. (2002). Feeding a world of 10 billion people: The miracle ahead. In Vitro Cellular and Developmental Biology – Plant, 38, 221–228.

[sum12503-bib-0005] Bourdin, F. , Sakrabani, R. , Kibblewhite, M. G. , & Lanigan, G. J. (2014). Effect of slurry dry matter content, application technique and timing on emissions of ammonia and greenhouse gas from cattle slurry applied to grassland soils in Ireland. Agriculture, Ecosystems and Environment, 188, 122–133.

[sum12503-bib-0006] Butler, T. J. , Biermacher, J. T. , Kering, M. K. , & Interrante, S. M. (2012). Production and Economics of Grazing Steers on Rye‐Annual Ryegrass with Legumes or Fertilized with Nitrogen. Crop Science, 52, 1931–1939.

[sum12503-bib-0007] Conway, G. , & Toenniessen, G. (1999). Feeding the world in the twenty‐first century. Nature, 402, C55. 10591226 10.1038/35011545

[sum12503-bib-0008] COSMOS UK (2017). Daily and sub‐daily hydrometeorological and soil data (2013–2015) [COSMOS‐UK]. NERC Environmental Information Data Centre. Retrieved from 10.5285/cdcf6ec3-1949-4fe7-a6f2‐

[sum12503-bib-0009] Crotty, F. V. , Fychan, R. , Scullion, J. , Sanderson, R. , & Marley, C. L. (2015). Assessing the impact of agricultural forage crops on soil biodiversity and abundance. Soil Biology and Biochemistry, 91, 119–126.

[sum12503-bib-0010] DEFRA (2010). Fertiliser Manual (RB209). In: *Fertiliser Manual*. (ed DEFRA), The Stationery Office (TSO), Norwich, pp. 257.

[sum12503-bib-0011] DEFRA (2016). Agriculture in the United Kingdom. In: *Agriculture in the United Kingdom* London, pp. 110. www.gov.uk/government/statistics/agriculture-in-the-united-kingdom-2016 Last accessed 8 March 2019

[sum12503-bib-0012] Dickinson, J. & Bonney, R. (Eds.). (2012). Introduction: Why Citizen Science? In Citizen Science: Public Participation in Environmental Research (pp. 1–304). Ithaca, NY: Cornell University Press.

[sum12503-bib-0013] Ellis, S. , Yamulki, S. , Dixon, E. , Harrison, R. , & Jarvis, S. C. (1998). Denitrification and N2O emissions from a UK pasture soil following the early spring application of cattle slurry and mineral fertiliser. Plant and Soil, 202, 15–25.

[sum12503-bib-0014] Fazey, I. , Evely, A. C. , Reed, M. S. , Stringer, L. C. , Kruijsen, J. , White, P. C. L. , … Trevitt, C. (2013). Knowledge exchange: A review and research agenda for environmental management. Environmental Conservation, 40, 19–36.

[sum12503-bib-0015] Frame, J. (1992). Seasonal Objectives and Management. In: Improved Grassland Management. The Farming Press, Ipswich, pp. 351.

[sum12503-bib-0016] Garnett, T. , Appleby, M. C. , Balmford, A. , Bateman, I. J. , Benton, T. G. , Bloomer, P. , … Godfray, H. C. J. (2013). Sustainable intensification in agriculture: Premises and policies. Science, 341, 33–34. 23828927 10.1126/science.1234485

[sum12503-bib-0017] Hanks, G. R. (2003). Narcissus and Daffodil: The genus Narcissus. Boca Raton, FL: CRC Press.

[sum12503-bib-0018] Horrocks, C. A. , Dungait, J. A. J. , Heal, K. V. , & Cardenas, L. M. (2014). Comparing N2O fluxes from recently created extensive grasslands and sites remaining under intensive agricultural management. Agriculture, Ecosystems and Environment, 199, 77–84.

[sum12503-bib-0019] Ingram, J. (2013). Farmer‐Scientist Knowledge Exchange In Thompson P. B., & Kaplan D. M. (Eds.), Encyclopedia of food and agricultural ethics (pp. 1–8). Netherlands, Dordrecht: Springer.

[sum12503-bib-0020] Kingsbury, N. (2013). Daffodil: The remarkable story of the world's most popular spring flower. Portland, OR: Timber Press.

[sum12503-bib-0021] Kloppenburg, J. (1991). Social‐theory and the de/reconstruction of agricultural science ‐ local knowledge for an alternative agriculture. Rural Sociology, 56, 519–548.

[sum12503-bib-0022] Krzywoszynska, A. (2016). What farmers know: Experiential knowledge and care in vine growing. Sociologia Ruralis, 56, 289–310.

[sum12503-bib-0023] McKinley, D. C. , Miller‐Rushing, A. J. , Ballard, H. L. , Bonney, R. , Brown, H. , Cook‐Patton, S. C. , … Soukup, M. A. (2017). Citizen science can improve conservation science, natural resource management, and environmental protection. Biological Conservation, 208, 15–28.

[sum12503-bib-0024] Morris, C. (2016). Environmental Knowledges and Expertise, International Encyclopedia of Geography.

[sum12503-bib-0025] Moser, C. A. , & Kalton, G. (2017). Survey methods in social investigation. London, UK: Taylor & Francis.

[sum12503-bib-0026] Murray, P. J. , Crotty, F. V. , & Van Eekeren, N. (2012). Management of grassland systems, and soil and ecosystem services In Wall D. H., Bardgett R. D., Behan‐Pelletier V., Herrick J. E., Jones T. H., Ritz K., Six J., Strong D. R., & Van der Putten W. H. (Eds.), Soil ecology and ecosystem services (pp. 282–293). Oxford, UK: Oxford University Press.

[sum12503-bib-0027] Polidoro, B. , & Clement, C. (2018). Beyond citizen science: Multigenerational education and mentoring in environmental monitoring—A case study. Integregrated Environmental Assessment and Management, 14, 521–522. 10.1002/ieam.4043 29906356

[sum12503-bib-0028] Pollard, G. , Roetman, P. , & Ward, J. (2017). The case for citizen science in urban agriculture research. Future of Food‐Journal on Food Agriculture and Society, 5, 9–20.

[sum12503-bib-0029] Rees, A. R. , & Hanks, G. R. (1996). Flowering date variation in narcissus. New Plantsman, 3, 244–248.

[sum12503-bib-0030] Soil Survey of England and Wales (1983). Soil Map of England and Wales, Scale 1:250,000. Soil Survey, Harpenden, UK.

[sum12503-bib-0031] Šūmane, S. , Kunda, I. , Knickel, K. , Strauss, A. , Tisenkopfs, T. , Rios, I. d. I. ,… Ashkenazy, A. (2018). Local and farmers’ knowledge matters! How integrating informal and formal knowledge enhances sustainable and resilient agriculture. Journal of Rural Studies, 59, 232–241.

[sum12503-bib-0032] Thomas, G. (2014). Research Note: Greenhouse Gas Emissions. National Assembly for Wales Research Service. Retrieved from https://naturalresources.wales/media/679462/national-assembly-for-wales-research-note-greenhouse-gas-emissions.pdf

[sum12503-bib-0033] Turrini, T. , Dörler, D. , Richter, A. , Heigl, F. , & Bonn, A. (2018). The threefold potential of environmental citizen science ‐ Generating knowledge, creating learning opportunities and enabling civic participation. Biological Conservation, 225, 176–186.

[sum12503-bib-0034] Vogeler, I. , Lucci, G. , & Shepherd, M. (2015). An assessment of the effects of fertilizer nitrogen management on nitrate leaching risk from grazed dairy pasture. The Journal of Agricultural Science, 154, 407–424.

[sum12503-bib-0035] Vranken, L. , Avermaete, T. , Petalios, D. , & Mathijs, E. (2014). Curbing global meat consumption: Emerging evidence of a second nutrition transition. Environmental Science and Policy, 39, 95–106.

